# Percutaneous Drug Delivery to the Masticatory System: A Systematic Review and Analysis of Randomized Clinical Trials

**DOI:** 10.3390/biomedicines13123110

**Published:** 2025-12-17

**Authors:** Kacper Galant, Kalina Romańczyk, Maciej Chęciński, Kamila Chęcińska, Natalia Turosz, Iwona Rąpalska, Amelia Hoppe, Alicja Jakubowska, Dariusz Chlubek, Maciej Sikora

**Affiliations:** 1 Faculty of Medicine, Medical University of Lodz, Al. Kościuszki 4, 90-419 Łódź, Poland; kacpergalant.ld@gmail.com; 2 Department of Oral Surgery, Preventive Medicine Center, Komorowskiego 12, 30-106 Cracow, Poland; kalina.romanczyk@wp.pl (K.R.); iwona.rzasa@uj.edu.pl (I.R.); amelia.a.hoppe@gmail.com (A.H.); 3 National Medical Institute of the Ministry of the Interior and Administration, Wołoska 137 Str., 02-507 Wasaw, Poland; kamila.checinska@pimmswia.gov.pl (K.C.); natalia.turosz@pimmswia.gov.pl (N.T.); alicja.jakubowska@pimmswia.gov.pl (A.J.); sikora-maciej@wp.pl (M.S.); 4 Department of Maxillofacial Surgery, Hospital of the Ministry of the Interior and Administration, Wojska Polskiego 51, 25-375 Kielce, Poland; 5 Chair of Oral Surgery, Jagiellonian University Medical College, Montelupich 4, 31-155 Kraków, Poland; 6 Department of Biochemistry and Medical Chemistry, Pomeranian Medical University, Powstańców Wielkopolskich 72, 70-111 Szczecin, Poland; dchlubek@pum.edu.pl

**Keywords:** temporomandibular disorders, masticatory muscles, drug delivery systems, percutaneous administration

## Abstract

**Background:** Due to the high frequency of pain associated with the masticatory system, in addition to the use of physiotherapy, it is sometimes beneficial to introduce percutaneous pharmacology. This is an example of a non-invasive alternative to the traditional route of administration, which can be highly effective and limit the risk of many side effects. The aim of this study is to evaluate the efficacy and safety of transdermal drug application by patients with temporomandibular disorders (TMD). **Methods:** A review of randomized controlled trials retrieved from PubMed, ACM, BASE, Scopus, Cochrane, and Google Scholar on 23 October 2025 was performed using the PRISMA Checklist. Studies that evaluated transdermal drug administration for masticatory system-related conditions were included. Trials involving interventions such as muscle punctures or any systemic treatments were excluded. The revised Cochrane tool (RoB 2) was used to assess the risk of bias. A statistical analysis of the effect of such therapy on pain and its influence on changes in mandibular abduction was performed. **Results:** A total of nine articles meeting the inclusion criteria were included, with a total of 390 participants. The risk of bias assessment indicated that most articles were of low risk of bias (6/8), while two were assessed as “some concerns”. The review found a significant reduction in pain on the visual analog scale (VAS) for selected active substances (*p* < 0.05). The greatest pain reduction occurred when 1.46% cannabidiol was used. There are few results regarding the effect of this method on jaw opening, and the results obtained are comparable for the placebo group, which was related to the application method. **Conclusions:** The results of this systematic review are promising and indicate the probable effectiveness of this method, but due to some limitations, this topic requires further investigation.

## 1. Introduction

### 1.1. Background

The temporomandibular joints (TMJs), together with the surrounding musculoskeletal structures, form a complex functional unit responsible for mandibular movement. The system includes both the joint components and the major masticatory muscles, which can be broadly divided into muscles that open and close the oral cavity [1,2,3]. Neck muscles, including the suprahyoid and infrahyoid groups, are primarily responsible for mandibular abduction. The lateral pterygoid muscle also plays a key role in opening, supporting the translational phase in which the mandibular condyle advances onto the articular eminence, and contributes to protrusion and lateral movements [1,4]. Masseter, medial pterygoid, and temporal muscles elevate the mandible and stabilize the TMJs during function [2].

The term temporomandibular disorders (TMD) refers to a broad range of musculoskeletal conditions affecting the temporomandibular region [5]. They are characterized by muscle soreness, TMJ pain, or both structures being painful at the same time [5,6]. Pain in the TMJ area is relatively common and occurs in 10–36.1% of patients, affecting women more frequently than men [5,7,8,9]. Additionally, individuals with TMD may experience limited movement of the mandible, headaches, neck pain, and noises from the TMJ [5,6,10]. These conditions may continually bother the patient during function or after palpation, worsening their quality of life [5,6,11,12].

Due to the above-mentioned symptoms, a range of analgesics is used. In 1986, the WHO introduced the analgesic ladder for the appropriate pain management, which consists of three steps [13]. At the first step, for mild pain, nonsteroidal anti-inflammatory drugs in combination with acetaminophen or alone with or without the addition of adjuvant medications are recommended for a short period of time. If pain persists or becomes moderate, treatment moves to the second step, where weak opioids may be used. At the third step, for very severe pain, an additional very strong opioid is introduced, e.g., oxycodone or tapentadol [14,15].

Nonsteroidal anti-inflammatory drugs (NSAIDs) affect cyclooxygenase (COX), which is involved in converting arachidonic acid to prostaglandins. Inhibition of this enzyme is responsible for analgesic and anti-inflammatory effects, as well as side effects, including (1) prolonged bleeding time, (2) lack of protective effect of prostaglandins on the gastrointestinal tract, and, as a result, peptic ulceration and bleeding, (3) renal dysfunction, caused by abnormal renal blood flow, and (4) aspirin-induced asthma, which is developed by 5% of patients [16,17]. The first side effect mentioned above led to the development of selective COX-2 inhibitors. However, this group of drugs is not without its drawbacks, as it poses a risk of cardiovascular diseases and should not be used in patients at risk [16].

Pain management is a fundamental patient right [18]. For postoperative patients, it allows a faster recovery, reducing the risk of complications [19]. Improperly conducted analgesic therapy can lead to the progression of postoperative pain into chronic pain. Chronic pain in TMD often stems from the underlying disorder itself. However, improper management of chronic pain can lead to central sensitization and maladaptive functional patterns. This highlights the importance of supporting patients in managing pain, which leads to improved overall well-being. Due to the complex nature of chronic pain and the inextricable link to proper functioning, some authors consider pain as the fifth vital parameter, which is as important as appropriate pulse, blood pressure, core temperature, and respiration [20,21]. Therefore, professional treatment requires a multidimensional approach encompassing psychological, social, and biological aspects [18].

### 1.2. Rationale

The etiology of TMD is multifactorial. One of the main etiological factors is increased tension in the chewing muscles, called bruxism. There are several types of bruxism, including awake bruxism, which is the prolonged and repetitive teeth clenching when the patient is conscious. It has been shown to have a strong relationship with TMD, as it contributes to the overloading of the masticatory muscles and the TMJ [22,23,24,25]. Myalgia and parafunction are associated with the development of symptomatic myofascial trigger points [3].

A widely accepted qualification for diagnosing and assessing TMD was established in 1992 [26]. In 2014, a revised version called “Diagnostic Criteria for Temporomandibular Disorders (DC/TMD) for Clinical and Research Applications” was presented [27]. It includes myalgia classification and assessment tools. The most common symptoms of TMD are muscle pain and discomfort in nearby soft structures, which can radiate to the head or neck. The treatment of TMD aims to reduce pain and improve patients’ quality of life through a multidisciplinary approach using natural methods (acupuncture, compresses), psychological and behavioral programs, occlusal splint therapy, physiotherapy, pharmacotherapy, and, in some cases, surgical intervention. Medications used to treat TMD include analgesics, nonsteroidal anti-inflammatory drugs, anticonvulsants, muscle relaxants, benzodiazepines, hyaluronic acid, and glucosamine [28,29].

The subject of this study is the application of therapeutic agents specifically for pain in the masticatory muscles [30,31]. This systematic review is limited to topical agents, the least. Invasive method for delivering medications to the masticatory system. Although topical therapies are widely used clinically, the evidence supporting their effectiveness in TMD remains fragmented, with a clear lack of standardized reporting on core outcomes [32,33,34,35,36,37,38,39,40,41]. In particular, current literature provides inconsistent data on pain reduction, mandibular mobility, health-related quality of life, and adverse reactions, highlighting an important gap that warrants systematic evaluation [33,34,35,36,37,38,39,40,41].

### 1.3. Objectives

This systematic review aims to evaluate the efficacy and safety of percutaneous drug delivery methods in patients with temporomandibular disorders, with a focus on clinical outcomes. The primary outcome was pain reduction, while secondary outcomes included changes in mandibular mobility (mouth opening), health-related quality of life, and adverse reactions. We hypothesize that percutaneous drug delivery can provide clinically meaningful improvements in these parameters.

## 2. Methods

This systematic review was developed following the Preferred Reporting Items for Systematic Review and Meta-Analysis (PRISMA) methodology. Checklists for the report and its abstract can be found in the Supplementary Documents S1 and S2.

### 2.1. Eligibility Criteria

This article includes publications in which patients with TMJ disorders were treated with any type of medication to relieve pain. This medication was administered transdermally or transmucosally to the masticatory system. Jaw opening disorders were also considered. Jaw opening varies among populations; 40–52 mm is considered normal. Values below 40 mm were considered abnormal. The electronic searches were conducted using English-language keywords, and only studies published in English were considered eligible. Clinical trial registries were not searched, as the review was restricted to fully published randomized clinical trials rather than unpublished or registry-listed studies. Reference lists were not searched as part of the study identification process. Table 1 presents the eligibility criteria based on which the publications were assessed.

### 2.2. Data Sources and Search Strategy

Searches were conducted in the following databases: (1) ACM Guide to Computing Literature, (2) Bielefeld Academic Search Engine, (3) Cochrane, (4) Elsevier Scopus, and (5) National Library of Medicine PubMed. Grey literature was searched using Google Scholar. Final searches were conducted on 14 October 2024. An updated search was performed on 23 October 2025, before the publication of the review. No filters were used in the search engines. The same query, based on the eligibility criteria, was used for each of them (Appendix A).

### 2.3. Selection and Data Collection Processes

All found records were entered into the Rayyan tool (Version 2024-10-14, Qatar Computing Research Institute, Doha, Qatar, and Rayyan Systems, Cambridge, MA, USA), and a manual deduplication process was performed (K.R.). In the next step, two authors (K.R. and M.C.) screened titles and abstracts. Whenever inconsistencies were found when comparing the assessments of both researchers, the dubious reports were qualified for the full-text evaluation along with those that were unanimously eligible. The full-text assessment was done by the same pair of authors. In the event of disagreement at this stage, the decision was made by consensus among the entire team of authors.

Numerical data were manually extracted by two authors (K.G. and K.R.) and then tabulated into a spreadsheet using Google Workspace software (Version 2025-10-23, Google LLC, Mountain View, CA, USA). Disputes were resolved by consensus, with one author (M.C.) casting the deciding vote. When necessary, we contacted the corresponding authors to obtain missing data.

### 2.4. Data Items and Study Risk of Bias Assessment

The authors collected the following data from the source articles: (1) first author and year of publication; (2) diagnosis; (3) number of patients in the control and study groups; (4) structure to which the drugs were administered; (5) name of the drug; (6) volume, concentration of the drug or the active substance itself; (7) method of drug administration; (8) presence of additional activities (massage of the area of drug administration, muscle massage, iontophoresis, laser, heating of the body area). Each eligible study arm, whether experimental or control, was included in the analysis if it met the predefined inclusion criteria (Table 1).

In addition, the investigators collected the following data from the content of the primary study reports: (1) initial health-related quality of life; (2) subsequent health-related quality of life values; (3) initial pain intensity; (4) subsequent pain intensity values; (5) initial range of mandibular abduction; and (6) subsequent ranges of mandibular abduction.

Outcome data were extracted at the timepoints reported in the original studies, without imposing additional harmonization. It was assumed that a month consists of 4 weeks (28 days) to unify tabular syntheses and visualizations in charts. To ensure comparability across trials, all pain intensity measures were converted to a 0–10 scale, mandibular abduction values were expressed in millimeters, and quality-of-life outcomes were normalized to a 0–10 scale.

The risk of bias was assessed using the revised Cochrane tool for assessing the risk of bias in randomized trials (RoB 2) by two authors (K.G. and K.R.) and verified by another author (M.C.).

### 2.5. Effect Measures and Synthesis Methods

Mean difference (MD), standard error (SE), and 95% confidence interval (CI) were calculated to measure the effect. MedCalc Comparison of Means Calculator (version 23.0.6; MedCalc Software Ltd., Ostend, Belgium) and Practical Meta-Analysis Effect Size Calculator (version 2023.11.27; Campbell Collaboration, Philadelphia, PA, USA), and Statistica software (version 13, TIBCO Software Inc., Palo Alto, CA, USA) were used.

A quantitative synthesis would have been undertaken only if at least two randomized clinical trials evaluated the same intervention using comparable formulations, dosing regimens, outcome definitions, and timepoints; in such cases, a random-effects model would have been applied. However, no intervention met these criteria, and therefore no meta-analysis was performed.

### 2.6. Registration and Protocol

The protocol was prospectively published in PROSPERO (ID—CRD42025632021).

## 3. Results

### 3.1. Study Selection

**Figure 1:**
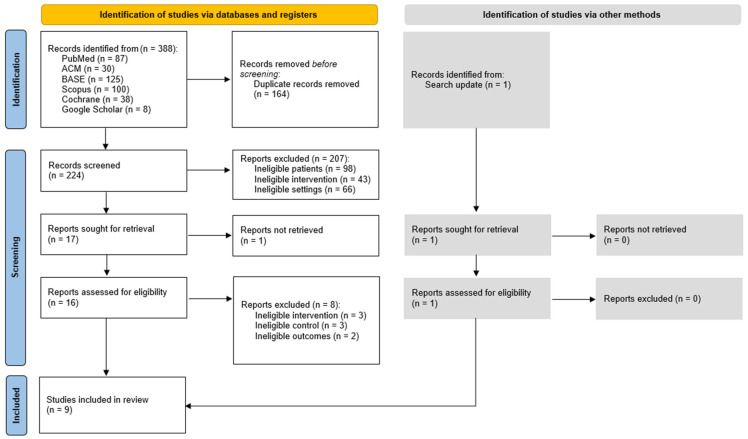
PRISMA flow diagram.

Database and grey literature searches yielded a total of 388 records, which were manually deduplicated and selected in screening stages. Details of the selection process are illustrated in Figure 1, which follows the PRISMA Flow Diagram standards. Reports rejected at the full-text review stage are listed in Table A1, along with the reasons for exclusion.

### 3.2. Study Characteristics

Nine clinical studies were included, each involving 15 to 68 participants diagnosed with myofascial pain, TMJ pain, TMD, or sleep bruxism. These patients underwent transdermal or transmucosal application of medications with potential anti-inflammatory and/or analgesic effects. Two studies also utilized phonophoresis. All studies also included a placebo control group. A total of 390 patients were included in the analysis, allowing for a preliminary analysis of the effectiveness of topical pain management methods within the masticatory system. The precise characteristics of the studies included in the review are presented in Table 2.

### 3.3. Risk of Bias in Studies

The Cochrane RoB 2 assessment indicates that the overall methodological quality of the included studies is generally high. Out of nine studies, six exhibited a low risk of bias across all assessed domains [33,34,37,38,39,41]. Three studies raised some concerns in one or two domains [35,36,40]. However, none were classified as having a high risk of bias. A comprehensive risk of bias analysis for the studies included in this systematic review is provided in Table A2.

### 3.4. Results of Individual Studies

Since no studies were excluded due to the risk of bias, all studies were included in syntheses. All of them described changes in the intensity of masticatory muscle pain between the first and last appointment (Table 3). Moreover, two described changes in mandibular abduction before and after the study (Table 4). Quality of life was only described by one author team, i.e., Winocur et al. [40]. They assessed the influence of the pain on their everyday life with the visual analog scale (VAS). They obtained the following value changes: 2.18 to 2.11 for the experimental group (capsaicin) and 3.34 to 3.23 for the control group (placebo), which indicates no significant effect.

Table 4 presents data on the change in jaw opening as a result of the interventions undertaken. Only two authors included such data in their articles. The largest increase in these values for the intervention group was obtained by Li et al. [38] from 31.38 (SD = 11.46) to 35.91 (SD = 11.45) for Ping-on.

Analysis of the incidence of adverse reactions indicates a wide variability in the tolerance of the substances used. No adverse reactions were reported in studies involving cannabidiol, aceclofenac, and indomethacin, confirming their high safety profile [33,34,35,41]. For the remaining substances, reported complaints were primarily mild to moderate local skin reactions. Application of capsaicin, Ping-On ointment, or Theraflex was most often associated with a burning sensation, warmth, skin redness, or eye irritation. It is worth noting, however, that in the case of capsaicin, the intensity of the burning sensation was so bothersome for some patients that it led to discontinuation of therapy. However, this is a predictable, not unexpected, effect and results from low individual patient tolerance. A separate risk was observed with the use of bee venom, which caused allergic reactions in sensitive individuals, including swelling and itching, requiring exclusion from the study. Allergic reactions are also variable and result from the random selection of patients. This situation could apply to any substance chosen. Despite the occurrence of the above-mentioned ailments, the lack of reports of serious systemic complications allows us to consider the use of these preparations as relatively safe (Table 5).

### 3.5. Results of Syntheses

**Figure 2:**
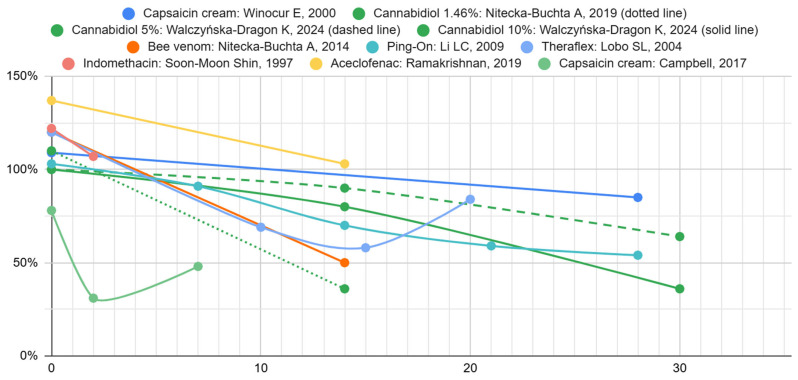
Study group pain scores as a percentage of control group pain scores over time [33,34,35,36,37,38,39,40,41].

The graphs in Figure 2 show the change over time in the effectiveness of pain relief of individual therapies in the study groups compared to the placebo groups. The differences on day “0” are due to different initial mean pain intensities between groups within one study. Further deviation of the curves from the initial values is due to the therapeutic effect of the active substances.

Table 6 presents the mean results obtained after 14–15 days of treatment for the intervention and placebo groups. The confidence intervals reported in Table 6 were calculated based on the number of subjects, the mean scores for the study and control groups, and their standard deviations. In the case of all data, they were analyzed, and statistical significance was assessed. The results were statistically significant in studies in which standard deviation values were available. Only in the case of aceclofenac was there no advantage of this substance demonstrated in comparison with the control group.

For mandibular abduction, Ping-On showed no significant difference compared with placebo (MD = 1.91 mm; 95% CI: −4.32 to 8.14). Capsaicin demonstrated a borderline, non-significant trend favoring placebo (MD = −7.0 mm; *p* ≈ 0.06). Overall, neither treatment produced a meaningful improvement in mouth opening.

## 4. Discussion

Significant differences in application method, the duration of the studies, tested agents, and numerous other factors preclude drawing definitive conclusions based on the included trials. Each study employed a distinct compound, which can be generally divided into three groups: (1) analgesic substances: aceclofenac gel, indomethacin gel, Theraflex-TMJ topical cream with methyl salicylate [35,39,41], (2) cannabidiol (CBD) [33,34], and (3) warming ointments: capsaicin cream, bee venom, Ping-On with peppermint oil and menthol [36,37,38,40]. Each of these preparations exerts a different therapeutic mechanism.

Analgesics exhibit anti-inflammatory effects, proven through many years of use, which involve inhibiting the production of COX by inhibiting the metabolism of arachidonic acid [42]. Their topical application limits undesirable effects in the form of digestive or cardiovascular system problems, depending on the affinity for a specific COX subtype [42,43].

Another substance used was CBD, which is a substance found in cannabis, classified as an organic compound [44]. Despite being an isomorph of THC and showing activity on the same pathway, it does not have psychoactive properties [45]. Many authors have demonstrated its antinociceptive and anti-inflammatory properties [46,47,48,49]. In addition, they have also been demonstrated with topical, not systemic, applications [50,51]. Although, referring to Pastina et al. [52], CBD is ineffective in reducing muscle pain when applied locally.

The final group comprises warming agents, representing a form of physiotherapy rather than traditional pharmacology. This approach carries a significant advantage due to minimal metabolic requirements and reduced side-effect risk. In addition, general warming of a given area can be easily applied in the case of pain, using, for example, hot compresses. It leads to muscle relaxation, pain reduction, and increased blood flow [53].

Another noteworthy aspect of individual studies is the variability in application techniques. Even the same substances were administered in other ways, for example, CBD [33,34]. In some cases, this may increase or eliminate the share of manual activities in pain reduction.

While some authors recommended the application of products only until complete absorption for several dozen seconds [34,39,40], others extended this period to several minutes with the use of circular movements as an additional massage [37,38], and others kept the preparation on the skin for up to 2 h [36]. Some of the researchers used the ultrasound technique [35,41].

However, the application used has visible side effects. In Ramakrishnan et al.’s study [35], the participants in the placebo group achieved a lower VAS score than those in the study group. The same situation occurred with the second author, who used the ultrasound method. The duration of Soon-Moon et al.’s study was much shorter, only 2 days. The baseline values in the case of both authors are not very similar for the placebo and study groups (6.40 vs. 4.68 and 6.03 vs. 4.96), which does not facilitate the analysis and makes it difficult to compare them rationally. Evidence indicates the effectiveness of ultrasound therapy as monotherapy in reducing pain [54]. However, some authors did not demonstrate such effectiveness but recommend considering its use in combination with other common modalities [55].

A comparison of the VAS values from day zero and after 14–15 days revealed a significant reduction in pain levels (*p* < 0.05) in studies providing standard deviations. The largest percentage reduction in the values was obtained by Nitecka-Buchta et al. [34]. These authors used CBD in a concentration of 1.46%. In contrast, another study by Walczyńska-Dragon et al. [33] employed higher concentrations of CBD (5% and 10%) but did not achieve a similar reduction in pain. This could probably be related to the method and place of application of the administered substances. The intergroup differences in the study by Walczyńska-Dragon et al. [33] indicate that higher concentrations of CBD produce a more substantial therapeutic effect. The imperceptible advantage of higher concentrations over lower ones may indicate a lack of a simple linear dose-effect correlation. The absence of this correlation may also indicate that lower concentrations are sufficient to achieve the desired therapeutic effects or that some pain components limit the benefit at higher doses and are insensitive to CBD. Alternatively, the observed differences may be due to random statistical factors between studies. Moreover, the interpretation of CBD dosing across studies is limited by inconsistent reporting of formulation strength. Although the Nitecka-Buchta et al. [34] formulation’s recipe could imply a 4% CBD content, the authors state that it contains ~1.46% CBD, which we adopted. The Walczyńska-Dragon et al. [33] study reports 5% and 10% gels, though with minor internal inconsistencies in per-dose amounts. These discrepancies, combined with the lack of pharmacokinetic data, prevent meaningful assessment of dose–response patterns and underscore the need for clearer formulation reporting in future trials.

After analyzing the reduction in pain intensity on the VAS, the next parameter assessed was mouth-opening range. Normal mouth opening varies among populations, but the critical functional value is 35–40 mm [26,56]. In case of unavailability of measurement devices, we can use three fingers of the patient (index, middle, and ring) for the assessment. The patient tries to open the mouth as much as possible and place these fingers in the mouth [57]. TMD is often accompanied by opening limitations and other occlusal disorders, such as catching and locking of the jaw [58]. Patients with TMJ disorders have a reduced range of motion in this joint compared to non-TMD patients [59]. Such disorders can be caused by muscle damage from dental injections, excessive chewing, yawning, and prolonged dental procedures, which lead to the development of myalgia, resulting in limited opening [58].

A detailed functional evaluation (mandibular abduction) was conducted by two research teams using warming agents [38,40]. Li et al. [38] obtained a greater increase in values for the Ping-On group, even though the duration of therapy was the same and amounted to 4 weeks. This may be related to the method of application, in which Li et al. [38] extended the application time to several minutes, compared to Winocur et al. [40], who recommended application only until complete absorption. Studies indicate the high effectiveness of physiotherapy in reducing trismus [60]. This is a probable reason for the better results obtained by one of the authors, indicating its greater contribution, which was also confirmed by reductions in the limitation of mandibular abduction in the case of placebo groups.

Given the promising effects observed in improving mandibular function and in reducing pain, it is also important to consider the broader context of local therapies for musculoskeletal pain. As indicated in the review by Derry et al. [61], local treatment can be as effective as oral treatment for acute musculoskeletal pain, with a significantly reduced risk of adverse effects. In another review by Derry et al. [62], the effectiveness of local therapy for chronic pain caused by osteoarthritis—a condition with similar characteristics to TMD—is emphasized. Therefore, this review fits perfectly into the treatment needs of patients with TMD but does not fill the gaps regarding other methods of local drug administration and modifications of the structures of drugs used, briefly discussed below.

Ionophoresis is a physiotherapeutic treatment using electrical current, which is used to introduce drugs into deep tissues [63]. The mechanism involves the introduction of a positively charged drug near the anode and moving within the electric field towards the cathode [64]. The best effects are elicited when the molecules are small and hydrophilic [65]. It is better than typical applications and delivers 10-2000 times more hydrophilic molecules to the skin [66]. Compared to other methods, iontophoresis has many advantages, such as safety, simplicity of use, and high transdermal efficiency [65]. It would be helpful to use this method, which has been known for decades, to maximize the effect of transdermal drug delivery to the masticatory system. Zuo et al. [67] demonstrated increased effectiveness of transdermal administration of NSAIDs such as ibuprofen and indomethacin (whose effect was studied by Soon-Moon Shin et al. [41]). This indicates both the gaps in the existing literature and the possibilities of using this method of drug administration in the treatment of TMD.

While ionophoresis is an advanced technique to improve transdermal delivery, it is essential to consider the broader principles and challenges related to transdermal drug administration. Transdermal drug administration has many advantages, including avoiding the first-pass effect, eliminating adverse effects from the gastrointestinal tract, and eliminating interactions with food or oral medications [68]. Due to the structure of the skin, lipophilic, nonpolar, and low molecular weight molecules (<500 Da) penetrate it to the greatest extent [68]. Even though the drug will present one of the above features, it may turn out to be insufficient, so there is a need to modify the structure, select the form, or use vesicles to maximize its effect. However, this route of administration may also have numerous disadvantages, such as allergic cutaneous reactions and localized irritation, which may cause systemic reactions and problems related to the time of drug penetration [69]. Continuous research is being conducted around the world on modifying the structures of selected substances to improve their therapeutic possibilities in the above method of administration.

An example of the modification of the structure of the introduced molecule is the use of prodrugs. In this technique, the drug is introduced in an inactive, more hydrophobic form [70], which makes it easier to overcome the skin barrier, and then undergoes metabolism and enzymatic conversion towards the active form [71]. Another method based on the modification of the drug structure is the use of vesicles. Depending on the main component, different types are used here, such as liposomes, ethosomes, transfersomes, niosomes, and phytosomes [68,72], Although nanovesicles have great potential, their use also has limitations, such as their instability, low drug loading, control of particle size, and short half-life [73]. Another goal of increasing drug absorption may be stratum corneum (SC) modification, which may increase their permeability [72]. This involves the use of occlusive dressings or absorption promoters [68]. Occlusive dressing and patches placed on the skin block the possibility of water evaporating from its surface and consequently increase the hydration of the SC. This method is particularly useful in the case of hydrophilic drugs. It also leads to increased blood flow in the vessels and an increase in temperature, which also results in increased permeability for drugs [70,74,75].

The carrier and type of preparation are also worth attention. As indicated by Derry et al. [61], the best formulation for administering analgesic medication in local therapy is a gel. In this form, diclofenac, ibuprofen, and ketoprofen have shown the greatest effectiveness.

Although promising results have been obtained, especially for CBD, current evidence does not allow for the formulation of strong clinical recommendations. Nevertheless, in the case of patients with contraindications to systemic therapy, local therapy and local application of drugs should be considered as one of the stages of multi-level pain treatment in TMD. The use of local therapy, which is a combination of selected substances with an appropriately selected method of application, is a safe and effective alternative to systemic treatment, especially in patients with contraindications to the use of NSAIDs, in the elderly, with diseases of the gastrointestinal tract or cardiovascular system. Such treatment increases the patient’s acceptability, reducing adverse effects.

Despite the lack of sufficient standardization of results, the review shows that the effectiveness of therapy can be enhanced by the appropriate pharmaceutical form, application technique, and individualization of treatment, as well as the proper selection of the active substance. The fact that different groups of drugs with varying mechanisms of action demonstrate the effectiveness of action indicates the possible personalization of treatment depending on the characteristics of symptoms and patient preferences. However, due to the methodological diversity of studies, their limitations, and the constant lack of clear guidelines, clinical decisions should be made individually based on the safety of the therapy and the doctor’s experience.

The search conducted in the presented review utilized an extensive query and integrated six databases, which is a positive aspect of the entire review and allows for a thorough assessment of the studied topic. No time limits were introduced either.

However, only the English-language literature was assessed, which could lead to the omission of studies from other regions of the world and a limitation of the available data. Due to the diversity of available data, direct comparison of results was not possible. Limitations stem from differences in treatment duration, drug administration methods, and the wide variety of substances used, including differences in pharmacodynamics, formulation, and mechanisms of action. This led to the inability to extend statistical analysis and the need to employ narrative analysis in selected cases.

The available clinical trials remain small, short in duration, and methodologically heterogeneous, with very limited documentation of safety outcomes. These issues further constrain the strength of the evidence and reinforce the need for more rigorous and standardized investigations.

Therefore, future studies should use standardized formulations, dosing methods, and outcome measures to enable clearer comparisons across treatments. Longer follow-up, systematic safety reporting, and trials directly comparing different drug classes and carriers are needed to identify the most effective and clinically meaningful approaches to percutaneous therapy in TMD.

## 5. Conclusions

Studies on cannabidiol, Theraflex, and Ping-On—each assessed as having a low risk of bias—showed short-term pain reduction compared with placebo, but none of the interventions produced a significant improvement in mandibular abduction. Quality-of-life outcomes were scarcely reported—only one study assessed this parameter and found no meaningful effect. Adverse reactions were mostly mild, local, and infrequent.

Overall, percutaneous drug delivery shows potential for modest short-term pain relief in temporomandibular disorders, but more rigorous and standardized clinical trials are needed to confirm its effectiveness and safety.

## Figures and Tables

**Table 1:** Eligibility criteria.

	Criteria for Inclusion	Criteria for Exclusion
Patients	Temporomandibular disorders	Cadaver and animal studies
Intervention	Local percutaneous or per-mucosal drug delivery to the masticatory system	Muscle punctures or any systemic intervention
Control	Any	None
Outcomes	Health-related quality of life, temporomandibular region pain, mandibular abduction	Non-quantifiable results
Timeframe	No limit	Not applicable
Settings	Randomized controlled trials	Preprints, non-English reports

**Table 2:** Study characteristics.

First Author, Publication Year	Entire Sample (*n*)	Diagnosis	Target Structure	Delivery Method	Control Group (*n*_0_)	Control Group Active Substance	Study Groups (*n*_1_, *n*_2_, etc.)	Study Active Substances
Walczyńska-Dragon K, 2024 [33]	40	Sleep bruxism and TMD	Masseter intraorally	Topical mucous application	20	Placebo formulation	20	5% and 10% cannabidiol formulations
Nitecka-Buchta A, 2019 [34]	60	Myofascial pain	Masseter	Topical skin application	30	Placebo ointment	30	1.46% cannabidiol
Ramakrishnan SN, 2019 [35]	50	TMJ pain	TMJ	Phonophoresis	25	Plain ultrasound	25	Aceclofenac gel
Campbell BK, 2017 [36]	15	TMD, TMJ pain	TMJ and masseter	Topical skin application	8	Placebo cream	7	8% capsaicin topical emollient cream base
Nitecka-Buchta A, 2014 [37]	68	Myofascial pain	Masseter	Topical skin application	34	Placebo ointment (vaseline)	34	0.0005% bee venom ointment
Li LC, 2009 [38]	55	Myalgia and/or TMJ pain	Masseter/Temporalis muscle/TMJ	Topical skin application	27	Placebo (vaseline)	28	Ping-On ointment
Lobo SL, 2004 [39]	52	Myalgia and/or TMJ pain	Masseter/TMJ	Topical skin application	26	Placebo cream	26	Theraflex-TMJ topical cream
Winocur E, 2000 [40]	30	Myalgia and/or TMJ pain	Masseter/Temporalis muscle/TMJ	Topical skin application	13	Placebo cream	17	0.025% capsaicin cream
Shin SM, 1997 [41]	20	TMJ pain	TMJ	Phonophoresis	10	Placebo gel	10	1% indomethacin gel

**Table 3:** Change in pain intensity at follow-up (measured on a 1–10 scale).

First Author, Publication Year	Patient Group	Data Type	Baseline Value	2 Days	7 Days	10 Days	14 Days	15 Days	20 Days	21 Days	28 Days	30 Days
Walczyńska-Dragon K, 2024 [33]	Cannabidiol 5%	Value	6.00				4.50					3.50
	Cannabidiol 10%	Value	6.00				4.00					2.00
	Placebo	Value	6.00				5.00					5.50
Nitecka-Buchta A, 2019 [34]	Cannabidiol 1.46%	Value	5.60				1.67					
		SD	1.38				1.44					
	Placebo	Value	5.10				4.60					
		SD	1.26				1.58					
Ramakrishnan SN, 2019 [35]	Aceclofenac	Value	6.40				2.12					
	Placebo	Value	4.68				2.06					
Campbell BK, 2017 [36]	8% capsaicin topical emollient cream base	Value	2.9	1.0	0.95							
	Placebo	Value	3.7	3.2	2.0							
Nitecka-Buchta A, 2014 [37]	Bee venom	Value	6.00				2.00					
	Placebo	Value	5.00				4.00					
Li LC, 2009 [38]	Ping-On	Value	4.94		4.25		3.15			2.51	2.19	
		SD	1.44		1.45		1.63			1.73	1.81	
	Placebo	Value	4.78		4.69		4.48			4.26	4.08	
		SD	1.19		1.31		1.24			1.28	1.44	
Lobo SL, 2004 [39]	Theraflex	Value	5.09			2.55		2.11	3.11			
		SD	2.44			2.40		2.14	2.76			
	Placebo	Value	4.25			3.70		3.66	3.71			
		SD	2.20			2.27		1.88	1.88			
Winocur E, 2000 [40]	Capsaicin cream	Value	7.25								3.39	
		SD	1.0								2.62	
	Placebo	Value	6.65								4.00	
		SD	2.32								2.50	
Shin SM, 1997 [41]	Indomethacin	Value	6.03	4.64								
		SD	1.49	1.21								
	Placebo	Value	4.96	4.35								
		SD	1.79	1.48								

SD—standard deviation.

**Table 4:** Change in mandibular abduction at follow-up (measured in millimeters).

First Author, Publication Year	Patient Group	Data Type	Baseline Value	28 Days
Li LC, 2009 [38]	Ping-On	Value	31.38	35.91
		SD	11.46	11.45
	Placebo	Value	33.18	34.00
		SD	12.39	12.07
Winocur E, 2000 [40]	0.025% capsaicin	Value	38.2	39.2
		SD	8.1	8.5
	Placebo	Value	43.2	46.2
		SD	8.4	10.4

SD—standard deviation.

**Table 5:** Adverse reactions reported in primary studies.

Author (Year)	Active Substance	Data on Adverse Reactions
Walczyńska-Dragon K, 2024 [33]	Cannabidiol	No adverse events reported.
Nitecka-Buchta A, 2019 [34]	Cannabidiol	No adverse reactions reported. No psychoactive side effects observed.
Ramakrishnan SN, 2019 [35]	Aceklofenac	No adverse reactions reported.
Campbell BK, 2017 [36]	Capsaicin	Pain: 1 patient in the experimental group and 1 non-TMD patient in an arm not included in this systematic review requested that the cream be removed due to excessive pain.
Nitecka-Buchta A, 2014 [37]	Bee venom	Allergic reactions: 4 people were excluded at the recruitment stage—1 in the experimental group and 3 in the control group. Risk of swelling, itching, and redness.
Li LC, 2009 [38]	Ping-On ointment	Eye irritation: 6 patients in the experimental group. Itching: 1 patient in the placebo group and 1 in the experimental group. Skin burning sensation: 1 patient discontinued treatment for 3 days. Adverse effects were mild in severity.
Lobo SL, 2004 [39]	Theraflex	Skin irritation and/or burning at the application site: observed in 2 patients in the experimental group and 2 in the placebo group.
Winocur E, 2000 [40]	Capsaicin	Heat sensation, burning sensation, and/or localized redness: 13 of the 15 patients reported mild to moderate symptoms; 2 patients discontinued due to an “unbearable” burning sensation; 2 patients in the placebo group reported a mild warm sensation and redness at the application site.
Shin SM, 1997 [41]	Indomethacin	No adverse reactions reported.

**Table 6:** Summary of evidence on topical treatments for masseter pain after 14–15 days versus placebo.

Substance Tested	Number of Studies	Number of Patients	Control Group	SD in Control Group	Study Group	SD in Study Group	Mean Difference	Standard Error	Lower 95% Confidence Interval	Upper 95% Confidence Interval	Significance Level	Risk of Bias
Aceclofenac	1	50	2.06	-	2.12	-	0.06	-	-	-	-	Some concerns
Bee venom	1	68	4.00	-	2.00	-	−2.00	-	-	-	-	Low
Cannabidiol 1.46%	1	60 (30/30)	4.60	1.58	1.67	1.44	−2.93	0.39	−3.71	−2.15	<0.0001	Low
Cannabidiol 5%	1	40	5.00	-	4.50	-	−0.50	-	-	-	-	Low
Cannabidiol 10%	1	40	5.00	-	4.00	-	−1.00	-	-	-	-	Low
Ping-On	1	55 (28/27)	4.48	1.24	3.15	1.63	−1.33	0.39	−2.11	−0.55	0.0012	Low
Theraflex	1	52 (26/26)	3.66	1.88	2.11	2.14	−1.55	0.56	−2.67	−0.43	0.0078	Low

## Data Availability

Data is contained within the article or Supplementary Material.

## Supplementary Materials

The following supporting information can be downloaded at: https://www.mdpi.com/article/10.3390/biomedicines13123110/s1, Supplementary Document S1: PRISMA 2020 Checklist; Supplementary Document S2: PRISMA 2020 for Abstracts Checklist.

## Abbreviations

The following abbreviations are used in this manuscript:

CBD	Cannabidiol
CI	Confidence Interval
COX	Cyclooxygenase
DC/TMD	Diagnostic Criteria for Temporomandibular Disorders
MD	Mean Difference
NSAID	Nonsteroidal Anti-Inflammatory Drug
PRISMA	Preferred Reporting Items for Systematic Review and Meta-Analysis
SC	Stratum Corneum
SD	Standard Deviation
SE	Standard Error
THC	Tetrahydrocannabinol
TMD	Temporomandibular Disorders
TMJ	Temporomandibular Joint
VAS	Visual Analog Scale

## Appendix A

**Search string**
(temporomandibular OR TMD OR TMJ) AND (muscular OR muscle OR masseter OR temporal OR temporalis OR pterygoid) AND (percutaneous OR transdermal OR per-mucosal OR transmucosal OR topical OR dermal OR mucosal OR epidermal OR surface OR delivery) AND (randomized OR random OR randomly)

**Table A1:** Excluded studies.

First Author, Publication Year	Title	Reason for Exclusion
Svensson P, 1997 [76]	Effect of systemic versus topical nonsteroidal anti-inflammatory drugs on post-exercise jaw-muscle soreness: a placebo-controlled study	Report not retrieved
L Di Rienzo Businco, 2004 [77]	Topical versus systemic diclofenac in the treatment of temporo-mandibular joint dysfunction symptoms	Ineligible intervention: systemic treatment
E C Ekberg, 1996 [78]	Diclofenac sodium as an alternative treatment of temporomandibular joint pain	Ineligible intervention: systemic treatment
Insha Azam, 2023 [79]	Effects of a program consisting of strain/counterstain technique, phonophoresis, heat therapy, and stretching in patients with temporomandibular joint dysfunction—PMC	Ineligible control: no control group
K Matsumoto, 2014 [80]	Local application of Aqua Titan improves symptoms of temporomandibular joint muscle disorder: a preliminary study	Ineligible control: no control group
Al-Zuheri, Insam, 2014 [81]	The Effect of Topical Anesthesia on Jaw Pain Thresholds in Patients with Generalized Pain	Ineligible outcomes: pressure pain threshold
Jerner, Adéle, 2014 [82]	The Effect of Topical Anesthesia on Pain Perception in Patients with Local Myalgia	Ineligible outcomes: pressure pain threshold
Schiffman EL, 1996 [83]	Temporomandibular joint iontophoresis: a double-blind randomized clinical trial	Ineligible outcomes: non-quantifiable results
Costa YM, 2020 [84]	Topical anesthesia degree is reduced in temporomandibular disorders patients: A novel approach to assess underlying mechanisms of the somatosensory alterations	Ineligible control: healthy volunteers

**Table A2:** Risk of bias in studies.

First Author, Publication Year	D1	D2	D3	D4	D5	Overall
Walczyńska-Dragon K, 2024 [33]	Low	Low	Low	Low	Low	Low
Nitecka-Buchta A, 2019 [34]	Low	Low	Low	Low	Low	Low
Ramakrishnan SN, 2019 [35]	Low	Some concerns	Low	Some concerns	Low	Some concerns
Campbell BK, 2017 [36]	Low	Low	Low	Low	Some Concerns	Some concerns
Nitecka-Buchta A, 2014 [37]	Low	Low	Low	Low	Low	Low
Li LC, 2009 [38]	Low	Low	Low	Low	Low	Low
Lobo SL, 2004 [39]	Low	Low	Low	Low	Low	Low
Winocur E, 2000 [40]	Low	Low	Some concerns	Low	Low	Some concerns
Shin SM, 1997 [41]	Low	Low	Low	Low	Low	Low
